# Supplementary material for: Modeling glioblastoma heterogeneity as a dynamic network of cell states
Source: Mol Syst Biol. 2021 Sep 16;17(9):e10105. doi: 10.15252/msb.202010105 (PMC8444284; doi:10.15252/msb.202010105)
Supplement: Supplementary file 6 — Source Data for Figure 5 [file MSB-17-e10105-s004.zip › Figure5A_sourcedata/GSEA_3017/hallmarks_stateD.GseaPreranked.1621934542601/HALLMARK_UV_RESPONSE_DN.html]

Details for gene set HALLMARK\_UV\_RESPONSE\_DN[GSEA]

|  || Dataset | state23017 |
| Phenotype | NoPhenotypeAvailable |
| Upregulated in class | na\_neg |
| GeneSet | HALLMARK\_UV\_RESPONSE\_DN |
| Enrichment Score (ES) | -0.34838137 |
| Normalized Enrichment Score (NES) | -1.5186949 |
| Nominal p-value | 0.053691275 |
| FDR q-value | 0.14933857 |
| FWER p-Value | 0.825 |
Table: GSEA Results Summary

  

Fig 1: Enrichment plot: HALLMARK\_UV\_RESPONSE\_DN      
 Profile of the Running ES Score & Positions of GeneSet Members on the Rank Ordered List

  

| PROBE | GENE SYMBOL | GENE\_TITLE | RANK IN GENE LIST | RANK METRIC SCORE | RUNNING ES | CORE ENRICHMENT || 1 | HAS2 |  |  | 4 | 0.596 | 0.0392 | No |
| 2 | MYC |  |  | 77 | 0.313 | 0.0056 | No |
| 3 | MMP16 |  |  | 132 | 0.269 | -0.0172 | No |
| 4 | INSIG1 |  |  | 150 | 0.258 | -0.0120 | No |
| 5 | PHF3 |  |  | 169 | -0.251 | -0.0082 | No |
| 6 | PIK3R3 |  |  | 277 | -0.261 | -0.0726 | No |
| 7 | ANXA4 |  |  | 333 | -0.267 | -0.0964 | No |
| 8 | AKT3 |  |  | 378 | -0.271 | -0.1113 | No |
| 9 | CDC42BPA |  |  | 415 | -0.275 | -0.1197 | No |
| 10 | GJA1 |  |  | 453 | -0.280 | -0.1285 | No |
| 11 | ATP2C1 |  |  | 505 | -0.286 | -0.1478 | No |
| 12 | SIPA1L1 |  |  | 527 | -0.288 | -0.1436 | No |
| 13 | DYRK1A |  |  | 551 | -0.291 | -0.1408 | No |
| 14 | ADD3 |  |  | 663 | -0.312 | -0.2047 | No |
| 15 | ANXA2 |  |  | 777 | -0.338 | -0.2684 | No |
| 16 | RND3 |  |  | 853 | -0.358 | -0.3011 | No |
| 17 | CDKN1B |  |  | 862 | -0.359 | -0.2818 | No |
| 18 | SYNE1 |  |  | 877 | -0.365 | -0.2668 | No |
| 19 | PDLIM5 |  |  | 941 | -0.390 | -0.2880 | No |
| 20 | VLDLR |  |  | 1004 | -0.416 | -0.3066 | No |
| 21 | PRKAR2B |  |  | 1005 | -0.416 | -0.2770 | No |
| 22 | LTBP1 |  |  | 1098 | -0.467 | -0.3152 | Yes |
| 23 | DLG1 |  |  | 1100 | -0.469 | -0.2827 | Yes |
| 24 | ATP2B1 |  |  | 1129 | -0.495 | -0.2693 | Yes |
| 25 | DDAH1 |  |  | 1132 | -0.497 | -0.2355 | Yes |
| 26 | SDC2 |  |  | 1200 | -0.580 | -0.2463 | Yes |
| 27 | SERPINE1 |  |  | 1218 | -0.617 | -0.2156 | Yes |
| 28 | COL5A2 |  |  | 1236 | -0.659 | -0.1820 | Yes |
| 29 | F3 |  |  | 1241 | -0.673 | -0.1374 | Yes |
| 30 | ID1 |  |  | 1252 | -0.703 | -0.0952 | Yes |
| 31 | PMP22 |  |  | 1302 | -1.010 | -0.0615 | Yes |
| 32 | IGFBP5 |  |  | 1307 | -1.052 | 0.0101 | Yes |
Table: GSEA details [plain text format]

  

Fig 2: HALLMARK\_UV\_RESPONSE\_DN: Random ES distribution      
 Gene set null distribution of ES for **HALLMARK\_UV\_RESPONSE\_DN**
